# Comparison of surgical and conservative therapy in older patients with distal radius fracture: a prospective randomized clinic al trial

**DOI:** 10.1186/s10195-024-00788-w

**Published:** 2024-10-01

**Authors:** David J. Haslhofer, Stefan M. Froschauer, Tobias Gotterbarm, Manfred Schmidt, Oskar Kwasny, Matthias Holzbauer

**Affiliations:** 1 Department for Orthopaedics and Traumatology, Kepler University Hospital GmbH, Johannes Kepler University Linz, Krankenhausstrasse 9, 4020 Linz and Altenberger Strasse 69, 4040 Linz, Austria; 2 Diakonissen Clinic Linz, Weissenwolffstrasse 15, 4020 Linz, Austria; 3 Department for Plastic and Reconstructive Surgery, Kepler University Hospital GmbH, Johannes Kepler University Linz, Krankenhausstrasse 9, 4020 Linz and Altenberger Strasse 69, 4040 Linz, Austria

**Keywords:** distal radius fracture, conservative therapy, surgical treatment, wrist surgery, palmar plate osteosynthesis

## Abstract

**Introduction:**

The distal radius fracture is considered the most common fracture in humans. For fractures classified as Arbeitsgemeinschaft für Osteosynthese (AO) 23-C1 + C2, there is no consensus on treatment in older patients due to inconsistent study results. The aim of this study was to compare conservative and surgical treatment in relation to wrist function and satisfaction in patients older than 65 years.

**Methods:**

In this prospective randomized clinical trial, patients aged older than 65 years who suffered an isolated AO-classified C1 or C2 distal radius fracture were randomized to surgical treatment using palmar plate osteosynthesis or conservative treatment. Patient-rated wrist evaluation (PRWE) score and disabilities of arm, shoulder, and hand (DASH) was assessed 3, 6 and 12 months post-interventionally. Satisfaction, range of motion (ROM) and pain scores were evaluated at 6 weeks and 3, 6 and 12 months post-interventionally.

**Results:**

A total of 80 patients with a mean age of 77.3 years (± 6.1 years) in the conservative group and 72.5 years (± 5.3 years) in the surgery group were included. Both the PRWE score, and the DASH score showed a statistically significant difference between the two groups after 3 months, 6 months and 12 months (*p* < 0.001). Patients in the surgical cohort showed a statistically significant higher satisfaction at the 6-week, 6-month and 12-month follow-up (*p* < 0.001 6 weeks + 12 months; *p* = 0.004 6 months).

**Conclusion:**

In this prospective randomized study, surgical treatment proved to be superior to conservative treatment in terms of the primary outcome variable PRWE score. Satisfaction was significantly better in the surgical group.

## Introduction

Due to ever-increasing life expectancy, the proportion of elderly people in our society is increasing. Therefore, the number of common injuries in this age group continues to increase, and their therapy becomes more and more important [1, 2].

Distal radius fracture (DRF) is considered one of the most common fractures in the elderly [1, 3]. While simple fractures are treated conservatively by plaster immobilization, complicated fractures require surgical therapy [4]. Advanced age is often associated with osteoporosis, diabetes and frailty and plays a pivotal role in fracture assessment and subsequent treatment decisions. With the development of surgical therapy using volar plating, anatomic reconstruction of unstable fractures can be approximated. In this regard, good long-term results concerning wrist function and pain have been shown [5].

For fractures of type 23-C1 + C2 according to the Arbeitsgemeinschaft für Osteosynthese (AO) classification, there is no consensus on therapy in older patients due to an inconsistent study situation. No clear therapy recommendation in favour of conservative or surgical treatment can be derived from the current literature [6–10].

The aim of our study is to compare conservative therapy and surgical therapy of 23-C1 + C2 DRF in terms of patient satisfaction and wrist function in patients older than 65 years.

The patient-rated wrist evaluation (PRWE) score is used as the main outcome variable, and the following null hypothesis has been formulated:

In patients older than 65 years with type 23-C1 + C2 DRF, conservative and surgical therapy show no significant difference in PRWE.

## Patients and methods

### Calculation

Prior to study initiation a sample size calculation was performed.

The standard deviation as well as the difference of the mean values of the patient-rated wrist evaluation (PRWE) score was derived from the publication by Walenkamp et al.[11].

Considering a Wilcoxon test with non-normal data distribution, an *α* of 0.05%, power of 80%, a difference in means of 11 points and a standard deviation of 14, which was published by Walenkamp et al. [11], a minimum of *n* = 31 patients in each group was required for this study. Thus, on the basis of the sample size calculations performed, including a drop-out rate of 30%, a case number of 40 patients per group was calculated.

The study was designed according to the Consolidated Standards of Reporting Trials (CONSORT) principles [12].

### Ethics

The study was approved by the local regional ethical committee.

### Patients

In a prospective manner, 80 patients older than 65 years who presented at our level-1 trauma centre between January 2021 and March 2022 with an isolated distal radius fracture AO classification type C1 or C2 were included in our study. Exclusion criteria were open fractures and absolute indication for surgery, pathologic fractures, refractures, patients with terminal illness, advanced dementia, pre-existing limitation of motion of the affected limb and patient preference against surgical treatment.

After confirmation through x-ray, every patient was treated by primary closed reduction under hematoma block anaesthesia and with a forearm cast. Study-specific informed consent was performed, and written patients’ consent was obtained. Patients were assigned to one of two groups (*n* = 40) – the surgery group and the conservative group – using permuted block randomization with blocks of 10 patients.

Pre-interventionally, the following data were collected: age, sex, dominant hand, classification of the fracture, side of injury, computed tomography (CT) assessment. Moreover, patient-rated wrist evaluation (PRWE) score and disabilities of arm, shoulder, and hand (DASH) score as well as range of motion (ROM) including dorsal extension, palmar flexion, ulnar deviation and radial deviation, and grip strength were assessed on the basis of the non-injured wrist.

### Conservative group

The treatment of the conservative group involved further immobilization with a forearm cast for 5–6 weeks, accompanied by regular follow-up examinations including x-ray controls. After cast removal, mobilization started through occupational therapy sessions.

### Surgery group

Patients assigned to the surgical group were treated with volar plate osteosynthesis after adequate decongestion, typically 1 week post-traumatically. A dorsal plaster splint was applied for another 2 weeks. After completed wound healing and suture removal, occupational therapy was started. Intraoperative data such as surgery date, implant, surgery duration, and complications were recorded.

### Follow-up regimen

Follow-up (f/u) was conducted in both groups at 6 weeks and 3, 6 and 12 months. During these assessments, patient satisfaction with wrist function was assessed using a five-item Likert scale (1 – very satisfied, 2 – satisfied, 3 – neither satisfied nor dissatisfied, 4 – dissatisfied, 5 – not satisfied at all), range of motion, grip strength and pain measured by a visual analogue scale (VAS) ranging from 0 to 10. At 3, 6 and 12 months post-interventionally, patient reported outcome measures (PROMs) including PRWE score and DASH score were evaluated. Any complications that occurred were documented.

### Statistical analysis

Statistical analysis was performed using IBM SPSS Statistics 29.0 (Chicago, IL, USA). The evaluation included the planned 80 patients in the above-mentioned observation period. All demographic and pre-, intra-, and post-interventional data were considered.

Data are presented using standard methods of descriptive statistics: Metric parameters with normal distribution are presented as mean ± standard deviation (SD) and in the case of a non-normal distribution as median (interquartile range [IQR]). The primary hypothesis was tested using the unpaired *t*-test in the case of normal data distribution and the Mann–Whitney *U* test in the case of non-normal distribution. These two tests were used to compare metric variables. Ordinal parameters were investigated using the Mann–Whitney *U* test. Nominal parameters were compared using the chi-squared test. If the requirements of this test were not fulfilled, the Fisher’s exact test was performed.

A significance value of *p* < 0.05 was deemed to be statistically significant for assessing the primary hypothesis. Other *p*-values smaller than 0.05 indicate a statistically relevant difference between groups.

## Results

A total of 80 patients, comprising 67 female (83.6%) and 13 male (16.4%) patients, met the inclusion criteria. Age ranged from 65 to 87 years with a mean of 74.8 years.

Patients had a mean age of 77.3 years (± 6.1 years) in the conservative group and 72.5 years (± 5.3 years) in the surgery group (*p* < 0.001). Comparing the conservative and surgical groups, fracture type 23-C1 occurred in 24 patients (60.0%) versus 9 patients (22.5%), and fracture type 23-C2 in 16 patients (30.0%) versus 31 patients (77.5%; *p* < 0.001). Regarding the non-injured wrist, PRWE score was 0.8 (0–17) in conservatively treated patients and 0.8 (0–2.0) in the surgical group (*p* = 0.598). Mean pre-trauma DASH score was 0.5 in the surgical group and 1.7 in the conservative group (*p* = 0.216; Table 1).

**Table 1:** Demographic data

	Conservative group	Surgical group	*p*-Value
*n*	40	40	
Dropouts	2 (5%)	0 (0%)	
Age, years	77.3 (67–87)	72.5 (65–83)	< 0.001*
AO classification			< 0.001*
23-C1	24 (60%)	9 (22%)	
23-C2	16 (30%)	31 (78%)	
Pre-trauma PRWE score	0 (IQR 0)	0 (IQR 0)	0.598
Pre-trauma DASH score	0 (IQR 0)	0 (IQR 0)	0.216
ROM dorsal extension (non-injured side), degrees	50 (IQR 20)	45 (IQR 20)	0.964
ROM palmar flexion (non-injured side), degrees	35 (IQR 15)	40 (IQR 10)	0.033*
ROM ulnar deviation (non-injured side) in °	30 (IQR 10)	30 (IQR 15)	0.155
ROM radial deviation (non-injured side), degrees	15 (IQR 159)	25 (IQR 9)	0.004*
Grip strength (non-injured side), kg	17.3 (IQR 8.8)	20.3 (IQR 7.6)	0.009*

*IQR* interquartile range, *PRWE* patient-rated wrist evaluation, *DASH* disabilities of arm, shoulder and hand, *ROM* range of motion; *p < 0.05

bold = statistically significant

Drop-out rate was 2.5% (two patients). Both patients were part of the conservative group. One patient passed away before the 3 months’ follow-up. Another patient did not attend follow-up examinations after the 6 weeks, despite several attempts to contact her.

**Fig. 1:**
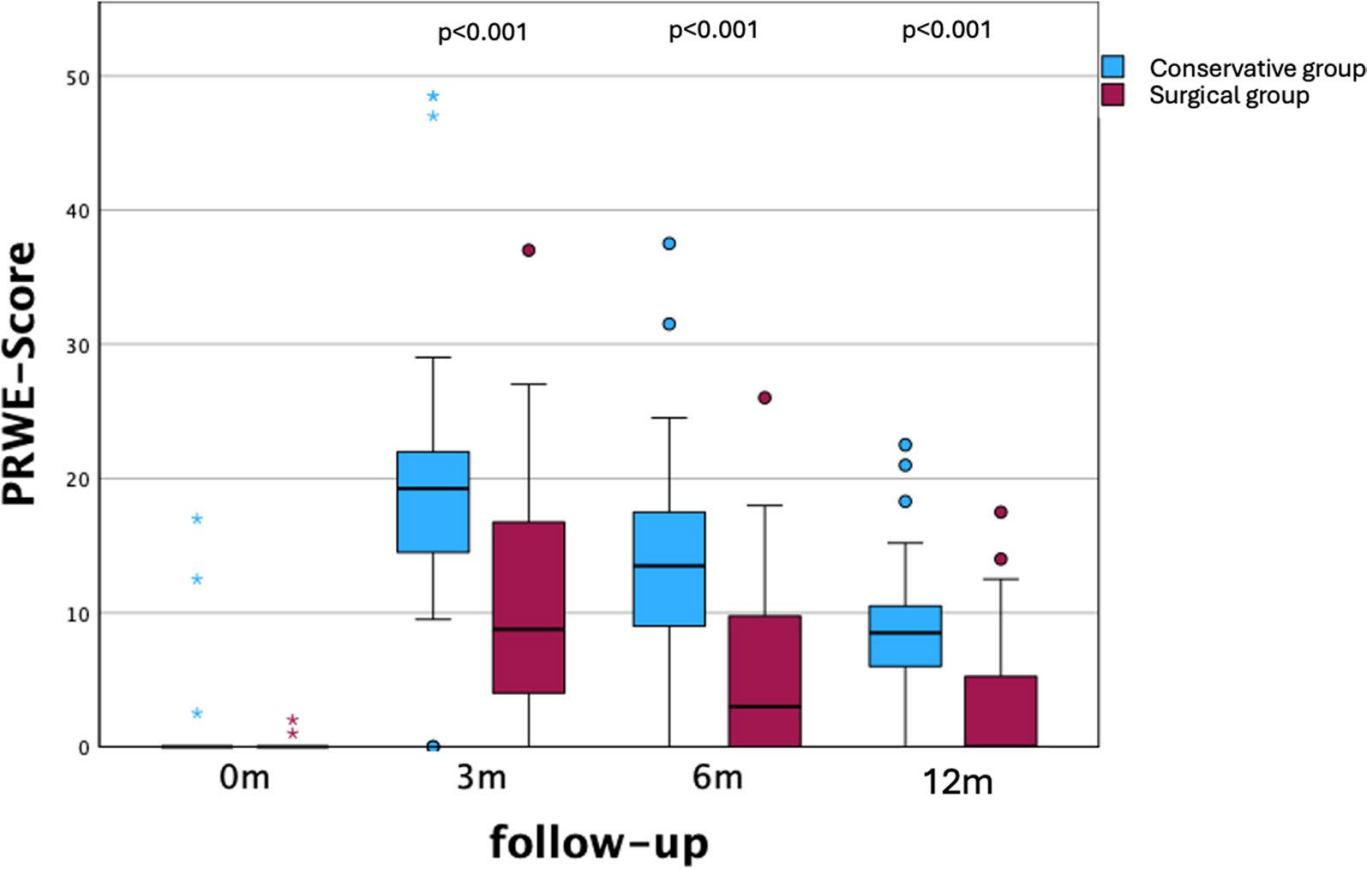
PRWE score pre-interventional and at follow-ups

**Fig. 2:**
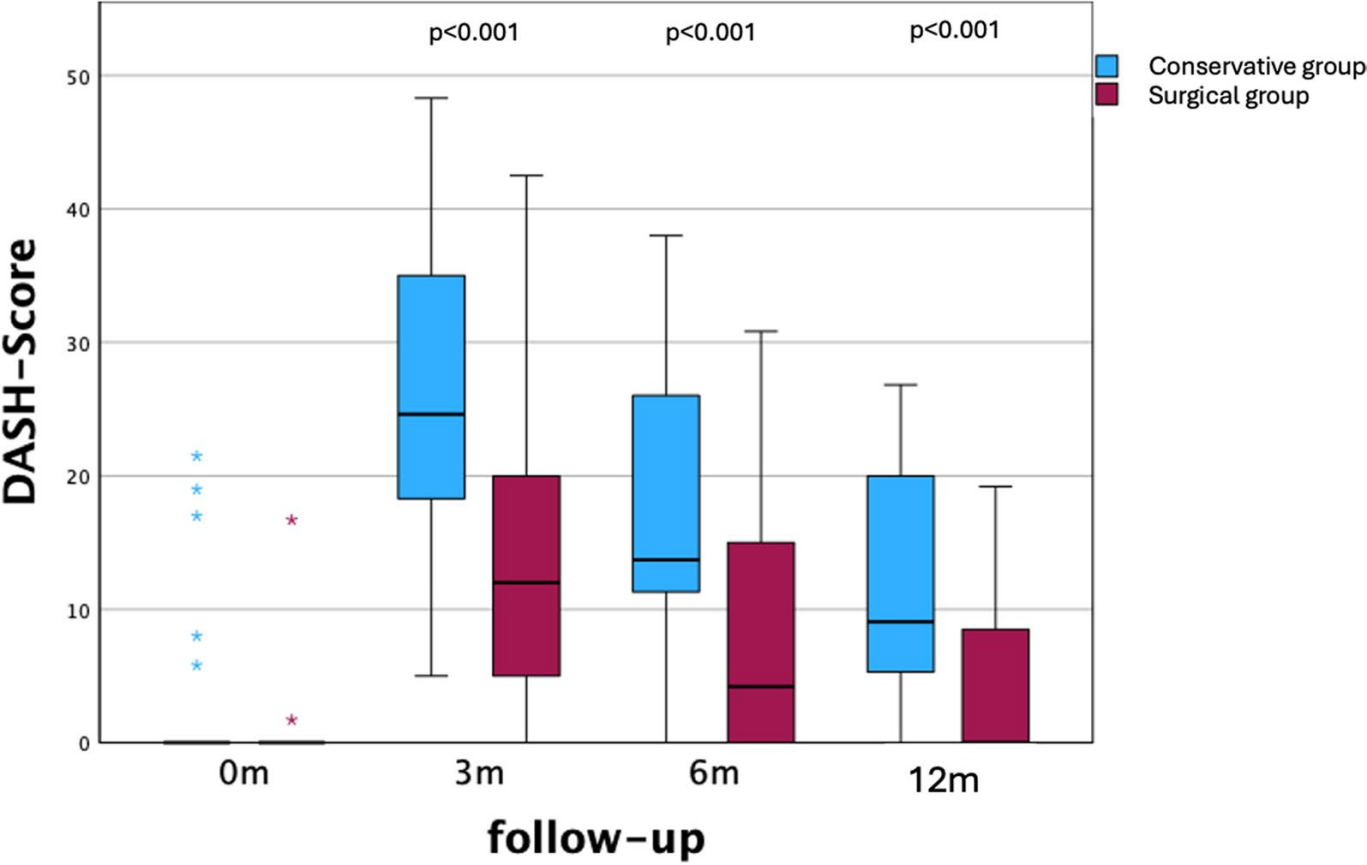
DASH score pre-interventional and at follow-ups

At 3, 6 and 12 months post-interventionally, the PRWE score as well as the DASH scores showed a significant difference between both groups (*p* < 0.001; Figs. 1 and 2**).**

Satisfaction was significantly higher in surgically treated patients at 6 weeks (*p* < 0.001), 6 months (*p* = 0.004), and 12 months (*p* < 0.001). There was no difference in pain between the two groups at all stages (Table 2**).**

**Table 2:** Post-interventional data

Follow-up		6 weeks		*p*-Value	3 months		*p*-Value	6 months		*p*-Value	12 months		*p*-Value
Group		Conservative	Surgical		Conservative	Surgical		Conservative	Surgical		Conservative	Surgical	
	Satisfaction (1–5)	2 (IQR 1)	2 (IQR 1)	< 0.001*	2 (IQR 2)	1 (IQR 1)	0.088	2 (IQR 1.3)	1 (IQR 1)	0.004*	1 (IQR 1)	1 (IQR 0)	< 0.001*
	VAS (pain) 0–10	2 (IQR 2)	2 (IQR 2)	0.115	0 (IQR 1.3)	0 (IQR 2)	0.987	0 (IQR 1)	0 (IQR 1)	0.546	0 (IQR 0.5)	0 (IQR 0)	0.739

*IQR* interquartile range, *VAS* visual analogue scale; *p < 0.05

bold = statistically significant

**Fig. 3:**
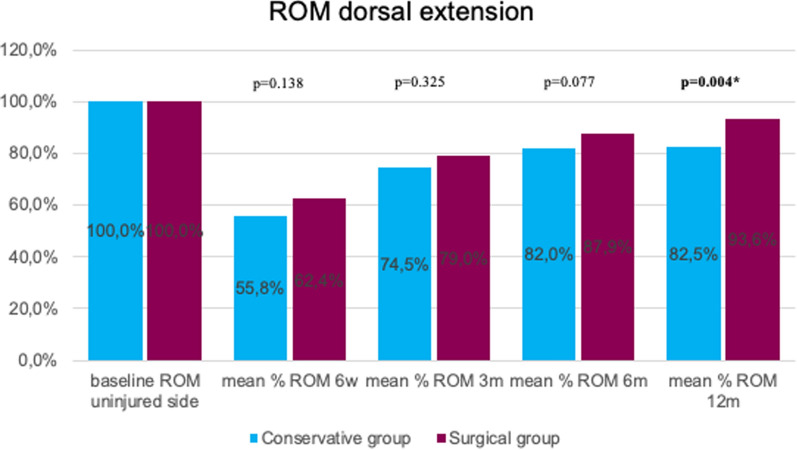
Mean percentage of ROM dorsal extension at follow-ups in reference to the baseline ROM of the uninjured side

**Fig. 4:**
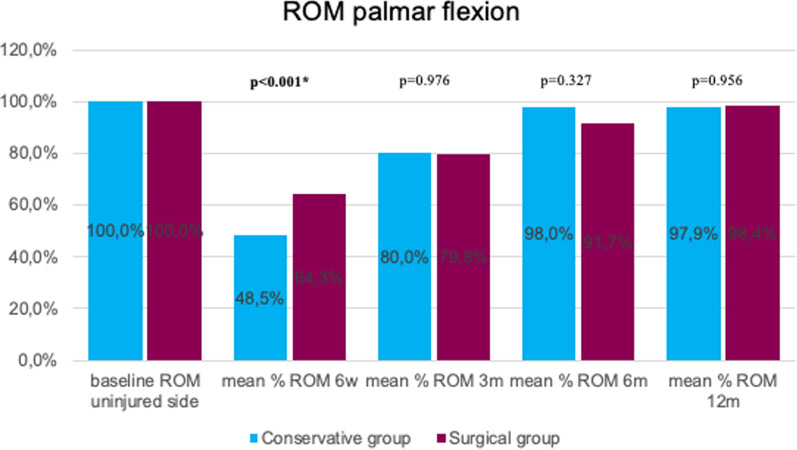
Mean percentage of ROM palmar flexion at follow-ups in reference to the baseline ROM of the uninjured side

**Fig. 5:**
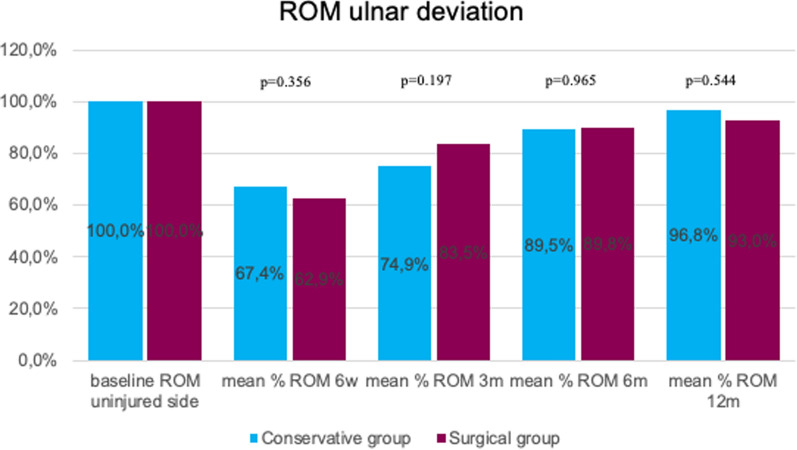
Mean percentage of ROM ulnar deviation at follow-ups in reference to the baseline ROM of the uninjured side

**Fig. 6:**
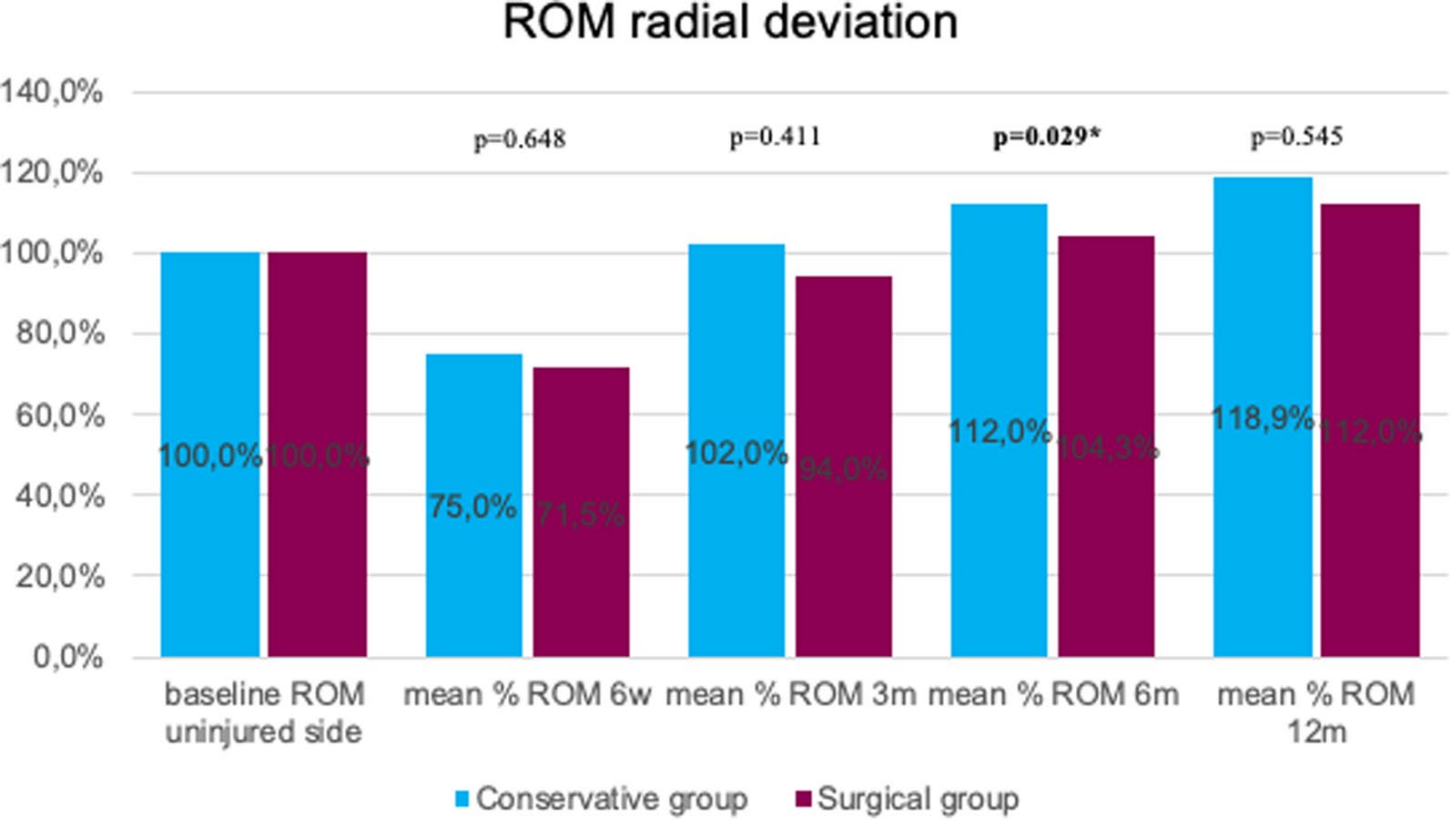
Mean percentage of ROM radial deviation at follow-ups in reference to the baseline ROM of the uninjured side

**Fig. 7:**
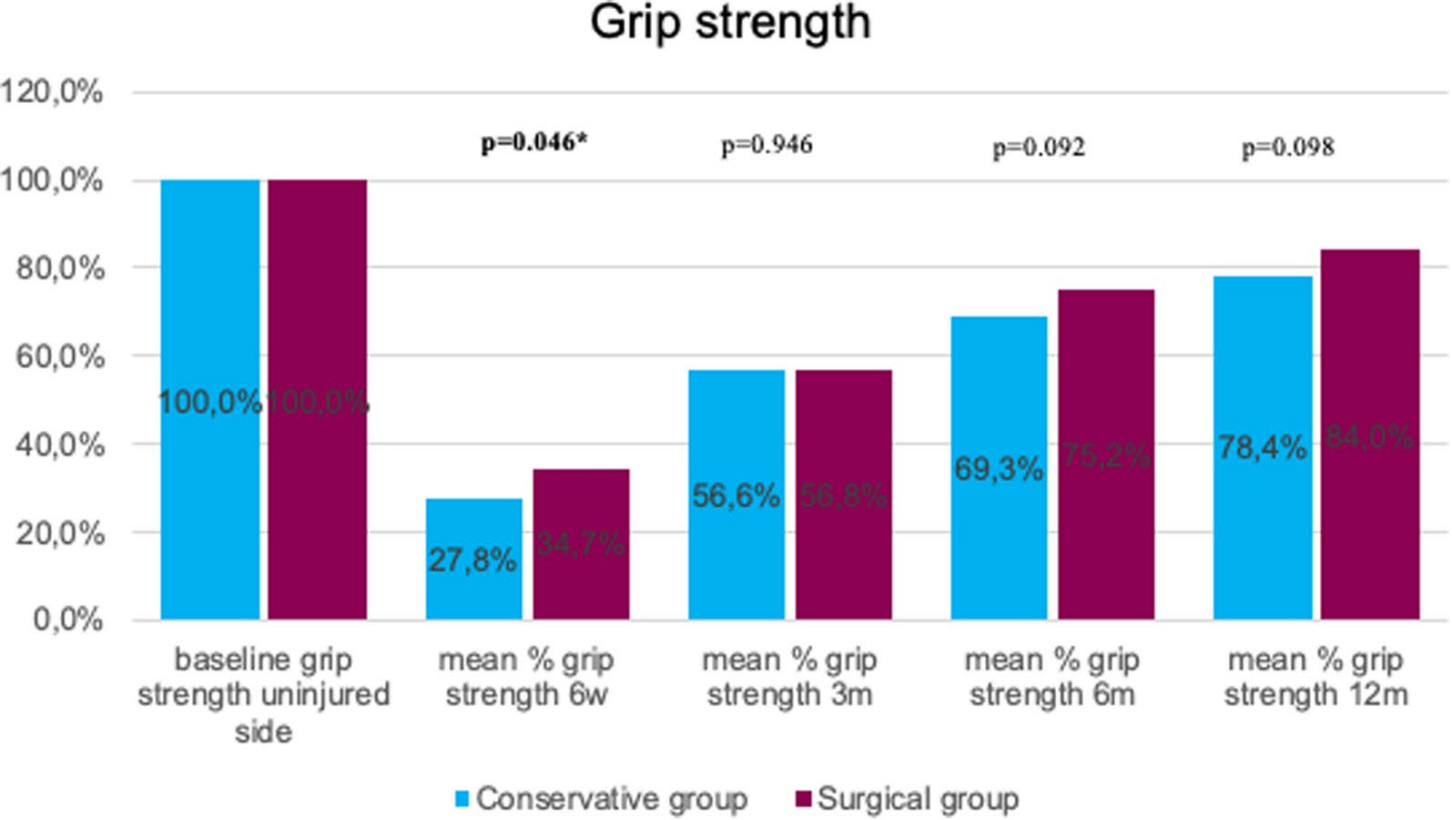
Mean percentage of grip strength at follow-ups in reference to the baseline grip strength of the uninjured side

Dorsal extension was significantly better at 12 months for surgically treated patients (*p* = 0.004). In the early stage (6 weeks), palmar flexion was significantly better in the surgical group (*p* < 0.001). No statistically significant difference between the two groups was detected regarding post-interventional ulnar deviation (ROM). At 6 months, radial deviation (ROM) was significantly better in the conservative group (*p* = 0.029). Grip strength was significantly lower in conservative patients at the 6-week follow-up (*p* = 0.046; Figs. 3, 4, 5, 6 and 7).

No complications occurred during surgery. No combined surgical procedure (i.e. k-wire or an additional screw) or an additional dorsal approach were necessary. Mean surgical time was 72 min (± 20 min). Five patients presented with post-operative complications (12.5%): Intra-articular screw position occurred in 3 patients (7.5%), and screw-loosening was seen in 1 patient (2.5%). All of them had revision surgery within 18 days. One patient developed carpal tunnel syndrome 3 months postoperatively (2.5%).

One patient in the conservative group presented ROM restriction of the forth and fifth finger after removing the forearm cast (2.5%), which could successfully be treated using occupational therapy. At 6-month examination two patients showed clinical signs of incipient carpal tunnel syndrome (5%), neither of whom wished for further diagnostic or therapeutic intervention. Two patients (5%) in the conservative group presented early (< 6 days) secondary displacement. Both were treated with a second closed reduction and no further displacement occurred during the conservative treatment.

None (0%) of all patients (surgical AND conservative groups) developed a post-interventional complex regional pain syndrome (CRPS).

## Discussion

The most important finding of this study is that patients older than 65 years with C1 or C2 distal radius fracture show better mid-term clinical and functional outcome when treated surgically. The null hypothesis of this study was rejected because the PRWE score showed significantly higher values in the surgery group compared with the conservative group.

There are a few prior randomized controlled studies comparing conservative and surgical treatment of DRF.

Regarding PROMs as the main outcome variable comparing these two treatment options, several previous authors arrive at divergent conclusions compared with our study. In 2011, Arora et al. found no difference of either PRWE or DASH score after 6 and 12 months [9]. Distinctly from our study, they also included patients with DRF of AO classifications A2, A3 and C3 in addition to C1 and C2 [9]. Similar results were published by Bartl et al., with no difference of the DASH score at 3 and 12 months post-interventionally [13]. The CROSSFIRE study group [14] as well as Tahir et al. [15] could not show any difference of the PRWE and DASH score after 3 and 12 months.

By contrast, Mulders et al. as well as Selles et al. [16] could present comparable results to our data in multicentre randomized control trials by showing significantly better PRWE and DASH scores at 3, 6 and 12 months post-interventionally in the surgical group [17]. However, both studies included patients aged between 18 and 75 years, which does not align with the age group in our study. In 2019, Saving et al. concluded in their study involving 140 patients with A2, A3 and C1–3 fractures who were older than 75 years that surgery results in superior PRWE and DASH scores at 3- and 12-months’ follow-up examination [18]. Hassellund et al. showed significantly better PRWE scores after 6 and 12 months as well as significantly better DASH scores after 3 and 6 months in the surgery group [3]. Even at the 2-year follow-up examination, both Martinez-Mendez [19] and Sirniö et al. [20] presented superior PRWE and DASH scores in patients treated with plate osteosynthesis compared with conservative treatment.

In 2022, Oldrini et al. published a systematic review and meta-analysis involving 12 randomized control trials (RCTs) that showed significantly better DASH scores in patients treated with palmar plate osteosynthesis after a short-term period of 3 months [21]. PRWE scores showed no significant difference after 3 and 12 months (*p* = 0.17 and *p* = 0.12) [21].

Alongside clinical scores, patient satisfaction is rendered as one of the most important parameters in any treatment. Apart from our study, Hassellund et al. [3] was – to the best of our knowledge – the only study that assessed patient satisfaction concerning the resulting function of their injured wrist. This study group corroborated our results by reporting superior satisfaction at 6 and 12 months in the surgical group [3].

While Martinez-Mendez et al. [19] and Tahir et al. [15] presented no significant difference concerning grip strength between conservatively and surgically treated patients, Arora et al. [9] and Saving et al. [18] published comparable outcomes to our data because they showed better grip strength in the surgical group. In this respect, the meta-analysis by Oldrini et al. again was not able to show any statistical significance [21].

Regarding complication rates, the literature reports a range between 8% and 39% in palmar plate osteosynthesis treatment [22] which aligns with the 12.5% post-operative complications observed in the surgery group.

One of the strengths of our study is its design as a prospective randomized trial as well as the study population of patients older than 65 years with an isolated AO-classified C1 or C2 DRF.

Nevertheless, there are a few limitations of our study. Firstly, the randomization process resulted in a difference in age and distribution of fracture subtypes between both cohorts. Furthermore, although every patient was referred to occupational therapy and was encouraged to exercise, we were not able to control and quantify the intensity. Although this was definitely not the focus of our study, another limiting factor is the missing assessment of correlation of radiological and functional outcomes.

## Conclusion

In this prospective randomized study, surgical treatment proved to be superior to conservative treatment in terms of the primary outcome variable, PRWE score. Satisfaction was significantly better in the surgical group.

## Data Availability

Not applicable.

## References

[1] Lofthus CM, Frihagen F, Meyer HE, Nordsletten L, Melhuus K, Falch JA (2008) Epidemiology of distal forearm fractures in Oslo, Norway. Osteoporosis Int 19(6):781–786 10.1007/s00198-007-0499-5 17985071

[2] Rupp MW, Nike; Pfeifer, Christian; Lang, Siegmund; Kerschbaum, Maximilian; Krutsch, Werner; Baumann, Florian; Alt, Volker. The incidence of fractures among the adult population of Germany – an analysis from 2009 through 2019. Dtsch Arztebl Int 2021; 118: 665–9; 10.3238/arzteblm20210238. 2021. 10.3238/arztebl.m2021.0238 PMC8727861 34140088

[3] Hassellund SS, Williksen JH, Laane MM, Pripp A, Rosales CP, Karlsen Ø et al (2021) Cast immobilization is non-inferior to volar locking plates in relation to QuickDASH after one year in patients aged 65 years and older: a randomized controlled trial of displaced distal radius fractures. Bone Joint J. 103b(2):247–255 10.1302/0301-620X.103B2.BJJ-2020-0192.R2 33517725

[4] Handoll HH, Huntley JS, Madhok R (2007) External fixation versus conservative treatment for distal radial fractures in adults. Cochrane Database Syst Rev. 10.1002/14651858.CD006194.pub2 17636832 10.1002/14651858.CD006194.pub2 PMC13061287

[5] Arora R, Gabl M, Gschwentner M, Deml C, Krappinger D, Lutz M (2009) A Comparative study of clinical and radiologic outcomes of unstable colles type distal radius fractures in patients older than 70 years: nonoperative treatment versus volar locking plating. J Orthop Trauma 23(4):237–242 19318865 10.1097/BOT.0b013e31819b24e9

[6] McQueen MM, Hajducka C, Court-Brown CM (1996) Redisplaced unstable fractures of the distal radius: a prospective randomised comparison of four methods of treatment. J Bone Joint Surg Br 78(3):404–409 8636175

[7] Beumer A, McQueen MM (2003) Fractures of the distal radius in low-demand elderly patients: closed reduction of no value in 53 of 60 wrists. Acta Orthop Scand 74(1):98–100 12635802 10.1080/00016470310013743

[8] Young BT, Rayan GM (2000) Outcome following nonoperative treatment of displaced distal radius fractures in low-demand patients older than 60 years. J Hand Surgery 25(1):19–28 10.1053/jhsu.2000.jhsu025a0019 10642469

[9] Arora R, Lutz M, Deml C, Krappinger D, Haug L, Gabl M (2011) A prospective randomized trial comparing nonoperative treatment with volar locking plate fixation for displaced and unstable distal radial fractures in patients sixty-five years of age and older. JBJS 93(23):2146–2153 10.2106/JBJS.J.01597 22159849

[10] Saving J, Severin Wahlgren S, Olsson K, Enocson A, Ponzer S, Sköldenberg O et al (2019) Nonoperative treatment compared with volar locking plate fixation for dorsally displaced distal radial fractures in the elderly: a randomized controlled trial. JBJS 101(11):961–969 10.2106/JBJS.18.00768 31169572

[11] Walenkamp MM, de Muinck Keizer RJ, Goslings JC, Vos LM, Rosenwasser MP, Schep NW (2015) The minimum clinically important difference of the patient-rated wrist evaluation score for patients with distal radius fractures. Clin Orthop Relat Res 473(10):3235–3241 26040969 10.1007/s11999-015-4376-9 PMC4562929

[12] Moher D, Hopewell S, Schulz KF, Montori V, Gøtzsche PC, Devereaux PJ, CONSORT et al (2010) explanation and elaboration: updated guidelines for reporting parallel group randomised trials. BMJ (Clin Res ed) 2010:340 10.1136/bmj.c869 PMC2844943 20332511

[13] Bartl C, Stengel D, Bruckner T, Gebhard F (2014) The treatment of displaced intra-articular distal radius fractures in elderly patients. Dtsch Arztebl Int 111(46):779–787 25491556 10.3238/arztebl.2014.0779 PMC4263909

[14] Lawson A, Naylor JM, Buchbinder R, Ivers R, Balogh ZJ, Smith P et al (2021) Surgical plating vs closed reduction for fractures in the distal radius in older patients: a randomized clinical trial. JAMA Surg 156(3):229–237 33439250 10.1001/jamasurg.2020.5672 PMC7807386

[15] Tahir M, Khan Zimri F, Ahmed N, Rakhio Jamali A, Mehboob G, Watson KR et al (2021) Plaster immobilization versus anterior plating for dorsally displaced distal radial fractures in elderly patients in Pakistan. J Hand Surg Eur 46(6):647–653 10.1177/1753193420977780 33487060

[16] Selles CA, Mulders MAM, Winkelhagen J, van Eerten PV, Goslings JC, Schep NWL (2021) Volar plate fixation versus cast immobilization in acceptably reduced intra-articular distal radial fractures: a randomized controlled trial. J Bone Joint Surg Am 103(21):1963–1969 34314402 10.2106/JBJS.20.01344

[17] Mulders MAM, Walenkamp MMJ, van Dieren S, Goslings JC, Schep NWL (2019) Volar plate fixation versus plaster immobilization in acceptably reduced extra-articular distal radial fractures: a multicenter randomized controlled trial. J Bone Joint Surg Am 101(9):787–796 31045666 10.2106/JBJS.18.00693

[18] Saving J, Severin Wahlgren S, Olsson K, Enocson A, Ponzer S, Sköldenberg O et al (2019) Nonoperative treatment compared with volar locking plate fixation for dorsally displaced distal radial fractures in the elderly: a randomized controlled trial. J Bone Joint Surg Am 101(11):961–969 31169572 10.2106/JBJS.18.00768

[19] Martinez-Mendez D, Lizaur-Utrilla A, de-Juan-Herrero J. Intra-articular distal radius fractures in elderly patients: a randomized prospective study of casting versus volar plating. J Hand Surg Eur 2018;43(2):142–7. 10.1177/1753193417727139 28870129

[20] Sirniö K, Leppilahti J, Ohtonen P, Flinkkilä T (2019) Early palmar plate fixation of distal radius fractures may benefit patients aged 50 years or older: a randomized trial comparing 2 different treatment protocols. Acta Orthop 90(2):123–128 30669897 10.1080/17453674.2018.1561614 PMC6461076

[21] Oldrini LM, Feltri P, Albanese J, Lucchina S, Filardo G, Candrian C (2022) Volar locking plate vs cast immobilization for distal radius fractures: a systematic review and meta-analysis. EFORT Open Rev 7(9):644–652 36125012 10.1530/EOR-22-0022 PMC9624483

[22] Quadlbauer S, Pezzei C, Jurkowitsch J, Rosenauer R, Pichler A, Schättin S et al (2018) Early complications and radiological outcome after distal radius fractures stabilized by volar angular stable locking plate. Arch Orthop Trauma Surg 138(12):1773–1782 30341694 10.1007/s00402-018-3051-5

